# Bioactive Paper Sensor Based on the Acetylcholinesterase for the Rapid Detection of Organophosphate and Carbamate Pesticides

**DOI:** 10.1155/2014/536823

**Published:** 2014-11-18

**Authors:** Mohamed E. I. Badawy, Ahmed F. El-Aswad

**Affiliations:** Department of Pesticide Chemistry and Technology, Faculty of Agriculture, Alexandria University, El-Shatby, Alexandria 21545, Egypt

## Abstract

In many countries, people are becoming more concerned about pesticide residues which are present in or on food and feed products. For this reason, several methods have been developed to monitor the pesticide residue levels in food samples. In this study, a bioactive paper-based sensor was developed for detection of acetylcholinesterase (AChE) inhibitors including organophosphate and carbamate pesticides. Based on the Ellman colorimetric assay, the assay strip is composed of a paper support (1 × 10 cm), onto which a biopolymer chitosan gel immobilized in crosslinking by glutaraldehyde with AChE and 5,5′-dithiobis(2-nitrobenzoic) acid (DTNB) and uses acetylthiocholine iodide (ATChI) as an outside reagent. The assay protocol involves introducing the sample to sensing zone via dipping of a pesticide-containing solution. Following an incubation period, the paper is placed into ATChI solution to initiate enzyme catalyzed hydrolysis of the substrate, causing a yellow color change. The absence or decrease of the yellow color indicates the levels of the AChE inhibitors. The biosensor is able to detect organophosphate and carbamate pesticides with good detection limits (methomyl = 6.16 × 10^−4^ mM and profenofos = 0.27 mM) and rapid response times (~5 min). The results show that the paper-based biosensor is rapid, sensitive, inexpensive, portable, disposable, and easy-to-use.

## 1. Introduction

The increasing concern for food pollution, due to the use of pesticides in agriculture, requires a strong effort in order to detect pollutants with reliable, economical, and rapid methods. Some pollutants like pesticides are very dangerous for human health. Therefore, there is widespread interest in the development of cost-effective, practical diagnostic tools that are amenable to rapid screening of specific target pesticide residues in foods and the environment [1–8]. Detection methods currently using liquid and gas chromatography are not suitable as rapid screening methods, as they are time consuming and provide only discontinuous analysis.

Recent research efforts focused on developing biosensors that can be incorporated into mobile detection devices. The biosensors are based on inhibition of specific enzymes by pesticides. In that respect, paper attracts considerable attention as a matrix for developing low cost analytical devices [9, 10]. Paper is affordable, abundant, and disposable and has high volume to surface ratio. Paper-based biosensors are usually fast-responding and low cost diagnostic tools in health and environmental applications. Bioactive papers are obtained by modification of paper matrix with biomolecules in order to add sensor functionality. One of the major advantages of bioactive paper sensors is that they are designed to operate without sophisticated equipment [11–16]. In bioactive paper sensors, enzyme-immobilized paper is the matrix for fluid sample transportation, biological detection, and the detection in a single step process. For paper-based biosensors, a variety of colorimetric formats have been developed including dipstick techniques and lab-on-paper microfluidic systems. These portable colorimetric biosensing papers could be extremely useful in remote settings or developing countries where simple bioassays are essential in the first stages of detecting disease and for monitoring environmental- and food-based toxins in the field [11, 12]. However, these bioactive paper sensors show promise and have utilized biorecognition elements that are physically adsorbed onto the paper surface, which can be of limited use in terms of retaining long-term bioactivity of fragile biomolecules such as enzymes.

Acetylcholinesterase (AChE) degrades the acetylcholine molecules into choline and acetic acid and organophosphate and carbamate pesticides are specific inhibitors of this enzyme. The toxicity of these pesticides depends on inhibition of AChE; thus the enzyme is a common bioevaluator for the detection of organophosphates and carbamates [17]. The inhibition of this enzyme has been proven to be useful in monitoring organophosphate and carbamate pesticides in different samples with various types of sensors including colorimetric [17, 18], surface plasmon resonance [19], electrochemical [20, 21], various nanomaterials-based methods [22–25]. In addition, paper matrix has been the improved material for some AChE-based detection devices such as sol-gel entrapment of gold nanoparticles for paper-dipstick sensor device [26] and a lateral flow application [15]. Moreover, microfluidic paper devices could be developed by patterning hydrophilic channels and hydrophobic barriers [27].

Therefore, our goal in the present study was to develop a rapid and cheaper method for pesticide residues detection. We report on a fully integrated paper-based sensor which employs biocompatible gel-derived chitosan as an entrapment agent to deposit not only the enzyme but also all other required reagents onto a paper support. As part of the optimization process, we investigated experimental conditions such as chitosan concentration, enzyme concentration, and enzyme substrate on the color output, as well as the effect of various pesticide concentrations on the sensitivity of the assay technique. In this case, inhibitors are first flowed directly into the AChE reaction area without exposure to the substrate (acetylthiocholine iodide, ATChI) by immersing the paper strip into the sample, and then the strip is immersed into ATChI to generate a yellow color. We show that the sensor can be used for rapid sensing of organophosphate and carbamate pesticides.

## 2. Materials and Methods

### 2.1. Chemicals and Reagents

Acetylcholinesterase (AChE) was obtained from the* Torpedo californica* electric organ; chitosan of low molecular weight (3.60 × 10^5^ Da and 89% degree of deacetylation), glutaraldehyde, Grade I, 25% in H_2_O, acetylthiocholine iodide 99% (ATChI), and 5,5′-dithiobis(2-nitrobenzoic) acid (DTNB) were purchased from Sigma-Aldrich Co. (USA). Profenofos as an organophosphate pesticide (purity of 93%) and methomyl as a carbamate pesticide (purity of 98%) were from the National Company for Agrochemicals and Investment, 473 El-Horreya St., Bolkly, Alexandria, Egypt. All commercially available solvents and reagents were of analytical grade and were used without further purification.

### 2.2. Solution Preparation

**Figure 1:**
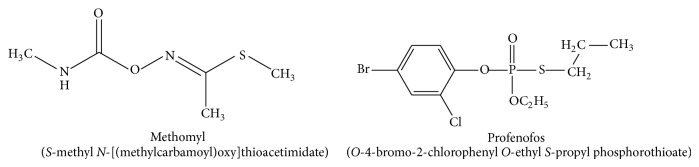
Molecular structures of methomyl and profenofos.

The AChE enzyme solutions were prepared freshly on the day of experiments in phosphate buffer (pH 7.0). Specific activity of the enzyme was calculated as nmoles ATChI hydrolyzed/mg protein/min. The concentration of DTNB is 10 mM in phosphate buffered solution (pH 8.0). The ATChI was prepared at concentration of 75 mM in distilled water. Stock solutions of chitosan (0.5, 1, 2, and 4%, w/v) were prepared by dissolving the weight of chitosan flakes in 10 mL of 0.5% aqueous acetic acid solution with stirring and the pH was adjusted to ≈6.0 using NaOH (0.1 M). Carboxymethylcellulose (CMC) and alginate solutions (0.5, 1, 2 and 4%, w/v) were prepared in distilled water. Stock solution of glutaraldehyde (0.25%) was made by diluting 100 *μ*L from commercial solution to one mL using distilled water. Pesticide stock solutions were made up daily and were not used for more than 3 h after preparation to minimize the degradation.  Figure 1 presents the molecular structures of methomyl and profenofos pesticides. Stock solutions were prepared by dissolution of the compounds with a sufficient volume of absolute ethanol, followed by dilution with distilled water to obtain the required concentration in mM.* Caution: both methomyl and profenofos pesticides are extremely toxic. These materials should be handled with gloves and masks and used in a fume hood.*

### 2.3. The* In Vitro* AChE Assay

**Figure 2:**
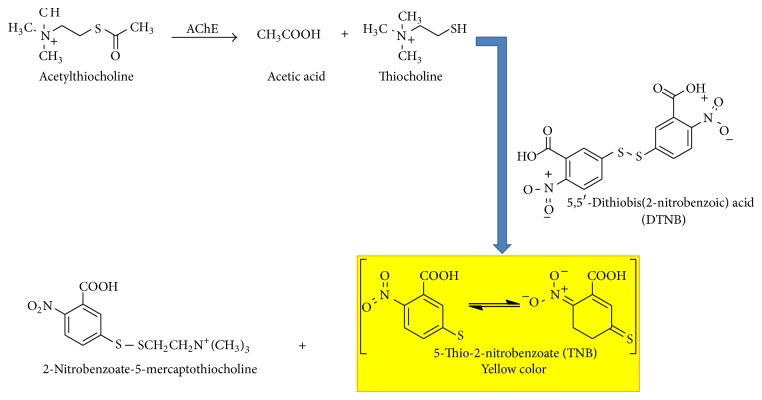
Acetylcholinesterase (AChE) hydrolyzes the acetylthiocholine and forms thiocholine base, which then reacts with dithiobisnitrobenzoate (DTNB) to generate 5-thio-2-nitrobenzoate (TNB, an anion), which is yellow in color.

AChE inhibition can be quantified by an assay method first described by Ellman et al. [28]. AChE hydrolyses ATCh to produce thiocholine base and acetate (Figure 2). Then, the free sulfhydryl group of thiocholine reduces DTNB to 5-thio-2-nitrobenzoate (TNB) which has the maximal absorbance at 412 nm. The difference between initial AChE activity and its after incubation with inhibitors is used to calculate the concentration of inhibitory molecules. Basically, the AChE inhibition by methomyl and profenofos assays was performed according to the following procedure: aliquots of 20 *μ*L from pesticide solution were incubated with 20 *μ*L AChE in a spectrophotometric cell containing 2.9 mL phosphate buffer (pH 8.0) and 50 *μ*L of DTNB (10 mM) solution for 10 min. Then 10 *μ*L of ATChI (75 mM) substrate solution was added and the absorbance was recorded at 412 nm. Assays in the absence of inhibitor and in the absence of enzyme-inhibitor (blank) served as control. The specific activity of AChE was expressed as nmoles of acetylthiocholine hydrolyzed/mg protein/min. Inhibition percentages of the activities against control were considered in the enzymatic assay.

### 2.4. Gel Material and Enzyme Immobilization Preparation

Chitosan, CMC, and alginate are biocompatible polymers and they are used as suitable matrixes for enzyme stabilization [29]. Gel mixtures of the biopolymer, AChE enzyme, buffer phosphate (pH = 8.0), glutaraldehyde as a crosslinking agent, DTNB as a chromophore, and glucose as a stabilizer were prepared for the indicated final concentrations in each experiment of the optimization process. The mixture was stirred for two min. These gels were used to prepare the biosensor as described below.

### 2.5. Fabrication of Bioactive Paper-Based Sensors

**Figure 3:**
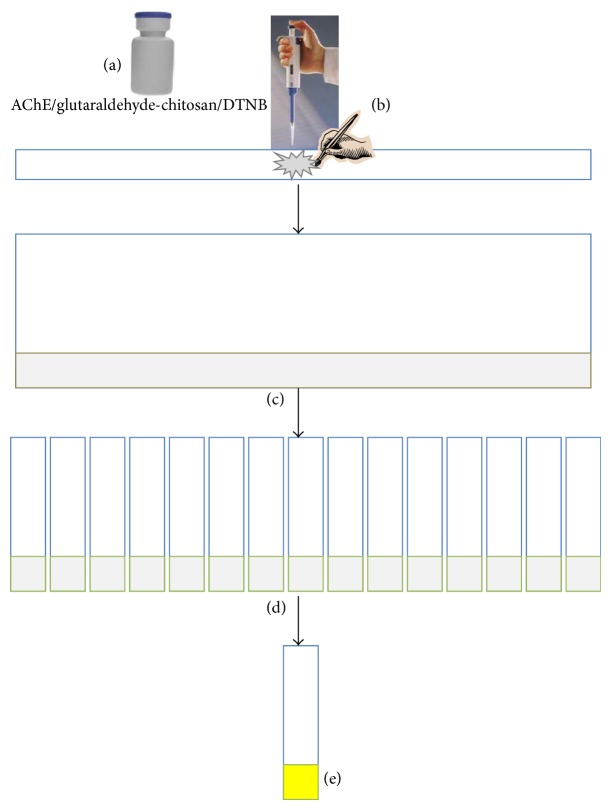
Schematic representation of biosensor support construction. (a) The gel formulation that contains AChE/glutaraldehyde-chitosan/DTNB mixture. (b) The AChE/glutaraldehyde-chitosan/DTNB was directly applied on Canson papers and dried at 37°C for 15 min. (c) The bioactive strip-based sensors were fixed on a Canson paper as a support. (d) Paper sheet was cut to 1 × 10 cm strips. (e) Paper strip after dipping in ATChI solution and incubation at 37°C for 5 min for yellow color formation.

For preparing biosensor strips, Canson paper was prepared in 1 × 30 cm strips and autoclaved at 120°C for 25 min prior to use. Paper strips were then fixed via two-sided plaster on Canson paper as supports. A mixture of chitosan gel immobilized and crosslinked with AChE enzyme by glutaraldehyde and DTNB was applied on the 1 × 30 cm strips paper by direct pipetting and spreading by painting brush and drying at 35°C for 15 min. The Canson paper as supports was cut into 1 × 10 cm that contained 1 × 1 cm piece of biosensor disk (Figure 3). For control experiments, a paper strip that did not contain AChE was made in the same manner.

### 2.6. Optimization of Bioactive Paper-Based Sensors

The effect of different parameters including concentration of chitosan, AChE, glutaraldehyde, DTNB, incubation time, the pH, and color intensity was examined to optimize the bioactive paper assay. The effect of three biopolymers including CMC, alginate, and chitosan was tested for gel formation. Different concentrations (0.5, 1, 2, and 4%) were initially investigated in phosphate buffer (pH = 8.0) and the suitable viscosity of the gel for immobilization technique was selected. Crosslinking process was optimized by adding different volumes (5–50 *μ*L) of 0.25% glutaraldehyde stock solution to the final volume of the gel 10 mL. For optimization of the AChE activity, different amounts (200–2000 *μ*L) from the crude enzyme extract (specific activity = 0.225 nmoles ATChI hydrolyzed/mg protein/min) were mixed with 10 mL of chitosan-glutaraldehyde/DTNB gel mixture and then were applied on the paper and dried at 35°C for 15 min. The optimum AChE activity was selected by observing the color intensity produced by the enzymatic hydrolysis of ATChI. DTNB was tested by adding 5–100 *μ*L of 10 mM stock solution to the gel matrix (10 mL) prior to applying on the paper strip. ATChI as a specific substrate of AChE was tested at 75 mM as the optimum concentration in the developing of the yellow color according to the method of Ellman et al. [28]. The performance of the paper sensor under optimized conditions was assessed directly by immersion of the paper strip into the ATChI solution (75 mM). The performance can be assessed by direct addition of substrate solution to the sensing area or by immersion of the paper strip into the substrate solution and incubation at 35°C for 5 min to allow the yellow color to develop without incubating the contaminated sample. In order to improve the stability of the enzyme AChE on the paper strip, glucose at 1, 3, 5, 10, and 15% (w/v) was tested according to Kavruk et al. [30]. The glucose was added in the gel mixture and final volume was always 10 mL. The performance of the sensor was essentially the same for both of these cases. However, in the case of direct analyte addition, only small amounts of ATChI were needed (5–10 *μ*L), relative to dipstick sensors (~2 mL), which will reduce the cost per assay.

### 2.7. Detection of Pesticides Using Paper-Based Biosensor

The inhibitory effects of methomyl (carbamate) and profenofos (organophosphate) pesticides were evaluated on the paper-based biosensor by measuring the decrease in the color intensity produced in ATChI based colorimetric assays. Color development change was converted to inhibitor concentration by visual comparison using methomyl and profenofos as inhibitors. The sensing area of the bioactive paper strip was first incubated with various concentrations of methomyl (6.16 × 10^−8^ to 0.62 mM) and profenofos (2.67 × 10^−6^ to 0.54 mM) solutions for 2 min following dipping in the ATChI (75 mM) solution and incubated at specific temperature of 37°C for 5 min to form yellow color. Up to 0.62 mM methomyl and 0.54 mM profenofos were investigated to determine the detection limit of the biosensor.

## 3. Results

### 3.1. AChE Immobilization and Biosensor Preparation

Immobilization of enzymes and other biologically active compounds onto biopolymers is a key element in using these structures for biosensing purposes. Fabricating biofunctionalized materials containing a high amount of the enzyme with high activity and stability is essential for the design of the biosensors. In the current study, we proposed an immobilization method that can be applied to the native AChE from electric eel, based on crosslinking between biopolymer chitosan molecules and glutaraldehyde on a paper. This method allows designing cheaper biosensor allowing the detection of organophosphate and carbamate pesticides. Initial studies on the effects of immobilization of AChE with chitosan, CMC, and alginate at 0.5, 1, 2, and 4% showed that chitosan is the best one at the concentration 2% (w/v). Therefore, CMC and alginate were not investigated further as viscosity modifiers and immobilizing agents. The high viscosity of the 2% (w/v) chitosan solution is likely due to the molecular weight of ca. 3.60 × 10^5^ Da. The optimum amount of the DTNB was 50 *μ*L DTNB (10 mM) to 10 mL of the chitosan gel (2%) prior to applying on the paper strip and fixed amount of phosphate buffer (1 mL, pH 8.0), AChE (1 mL), 200 *μ*L glutaraldehyde (0.25%), and 0.3 g glucose. This concentration of the DTNB resulted in a biosensor with more rapid color formation. Therefore, the overall results from these experiments resulted in the identification of the optimum concentrations of biosensor mixture as follows: 10 mL of chitosan gel (2%, w/v), 1 mL of phosphate buffer (pH = 8.0), 1 mL AChE, 200 *μ*L glutaraldehyde (0.25%), 50 *μ*L DTNB (10 mM), and then 0.3 g glucose. Using this sequence, and when the ATChI (75 mM) was used as substrate and incubation at 35°C for 5 min, the maximal color was obtained. The AChE activity on the bioactive paper strip was evaluated visually due to the color intensity produced by the enzymatic reaction according to Ellman's method. The performance can be assessed by immersion of the paper strip into the ATChI solution and incubation for 5 min at 37°C to allow the yellow color to develop. The long-term stability of AChE within the layered coating on the paper substrate was also investigated. Our results demonstrated that the enzyme retained >95% of its initial activity over a period of at least one month when stored at 4°C, indicating that both the enzyme and the DTNB reagent remained viable during storage. In order to improve the stability of the enzyme AChE on the paper strip, glucose at 1, 3, 5, 10, and 15% (w/v) was tested. The glucose was added in the AChE-DNTB mixture and final volume was always 10 mL.

### 3.2. *In Vitro* AChE Inhibition by Methomyl and Profenofos

**Figure 4:**
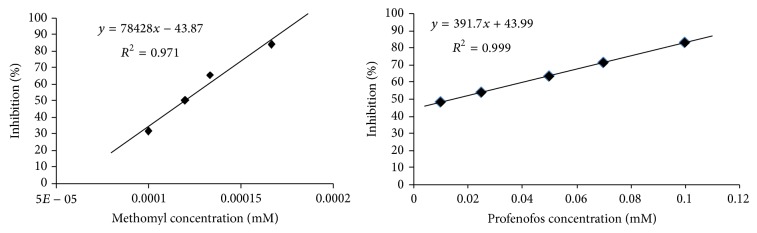
*In vitro* AChE inhibition by methomyl (carbamate) and profenofos (organophosphate) pesticides using Ellman et al. [28] method.

The data in Figure 4 showed that AChE was inhibited extensively by methomyl and profenofos and the inhibition was proportional to pesticide concentrations. However, methomyl was more potent pesticide than profenofos. The* in vitro* preincubation of 1.0 × 10^−4^, 1.2 × 10^−4^, 1.33 × 10^−4^, and 1.67 × 10^−4^ mM of methomyl caused significant dose dependent inhibition in AChE activity to 31.71, 50.62, 65.85, and 84.15%, respectively (Figure 4). However, the* in vitro* preincubation of 0.01, 0.025, 0.05, 0.07, and 1.0 mM of profenofos caused significant dose dependent inhibition in AChE activity to 48.04, 53.78, 63.34, 71.40, and 83.27%, respectively. This finding was confirmed by calculating the concentrations that caused 50% enzyme inhibition (I_50_) that were found to be 1.18 × 10^−4^ and 1.5 × 10^−2^ mM for methomyl and profenofos, respectively. In addition, the inhibition constant (*K*_*i*_) that were calculated from this equation *K*_*i*_ = [0.693/(I_50_ × time)] were found to be 978.81 and 7.7 mM for methomyl and profenofos, respectively. As well known that the *K*_*i*_ value is a measure of the affinity of the enzyme for the inhibitor [31], the present results confirm that methomyl is more potent in inhibition of AChE than profenofos.

### 3.3. Detection of Methomyl and Profenofos Using Paper-Based Biosensor Assays

In this study, a paper-based biosensor was developed as a rapid and reliable monitoring method for organophosphate and carbamate pesticides. The model interaction between methomyl and profenofos and their specific inhibitions of AChE were used to monitor quantitative performance of the biosensor. A biosensor for AChE inhibitory molecules has been developed by immobilizing the enzyme on a biopolymer chitosan through crosslinking mechanism by glutaraldehyde and DTNB as a chromophore agent in paper matrix.

To determine the working amounts of sensing components, a range of concentrations for each of them was systematically optimized for the best combination of AChE as the enzyme, DTNB as the chromophore, and ATChI as the artificial substrate. Optimum concentrations were selected according to the quantified color production and the observation based on the naked eye, since occasionally the proposed biosensor would be used in the field and/or at home with minimum instrumental help.

**Figure 5:**
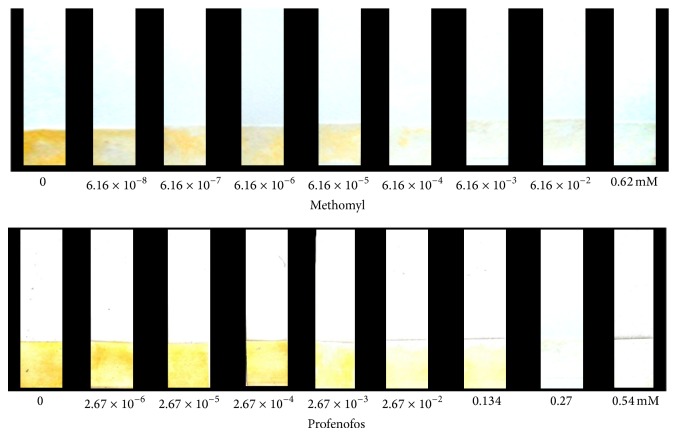
Relationship between inhibition of AChE activity and different concentrations of methomyl and profenofos using paper-based biosensor assays.

For evaluating the ability of developed biosensor for detection of AChE inhibitory effects, two commonly used inhibitors, methomyl and profenofos, were used. The biosensor response was tested by application of various concentrations of methomyl and profenofos samples. The untreated biosensor with inhibitor generally resulted in higher color development as expected after dipping in ATChI solution. Methomyl presence displayed high inhibition of AChE proportional to its concentration (Figure 5). The limit of detection of the biosensor was determined by testing the inhibitory effect of methomyl at a range of concentrations between 6.16 × 10^−8^ and 0.62 mM. Above 6.16 × 10^−4^ mM methomyl, the biosensor response to ATChI was completely inhibited. Thus, the limit of detection was determined to be 6.16 × 10^−4^ mM for methomyl. However, profenofos was performed at a range of concentrations between 2.67 × 10^−6^ and 0.54 mM. Above 0.27 mM profenofos, the biosensor response to ATChI was completely inhibited. Therefore, the limit of detection was determined to be 0.27 mM for profenofos.

## 4. Discussion

Detection of pesticide residues in foods and environmental samples is an important safety issue due to intensive agricultural applications and their consequent toxicity to consumers. Conventional methods of detection of pesticide residues rely on an analysis by gas and liquid chromatography with specific detection. Although these techniques are very powerful and can detect very low concentrations, they are still very expensive and require highly skilled personnel, expensive purification steps, and specialized major equipment [32]. In the last decades, new techniques based on biological detection have emerged. Among these techniques, biosensors have been shown to be very promising due to their simplicity and cost effectiveness compared to conventional techniques. Biosensors based on the inhibition of AChE have been intensively studied with the aim of detecting organophosphate and carbamate pesticides [15, 30, 33, 34].

In environmental monitoring, real-time detection of pollutants is important as sample conditions can fluctuate over the day and also over the time course of collection and transportation back to the lab. Thus, there is a real need for sensors which can allow on-site detection and accurate monitoring of environmental conditions. Paper sensing devices are a promising platform that, in principle, can be applied across a range of application areas such as health diagnostics, environmental monitoring, and food quality control. The paper itself has many advantages compared to plastic and glass substrates because it is low cost, disposable, abundant, and easy to transport. To date, researchers have focused on developing paper-based sensors with less complicated fabrication techniques and operation such that it can be used in developing world applications where simple and easy to operate devices are highly desirable [35–37].

In the current research, a paper-based biosensor was developed for the detection of organophosphate and carbamate pesticides. The biosensor strip is composed of dipstick paper sensor for analysis in a portable format to be suitable for detection of pesticide residues in filed and at home. The model interaction between two specific inhibitors (methomyl and profenofos) and their specific inhibition of AChE was used to monitor qualitative performance of the sensor. A biosensor for AChE inhibitory molecules has been developed by immobilizing the enzyme, its chromophore DTNB in paper matrix through crosslinking by glutaraldehyde and adsorption on the paper strip. The binding of inhibitors (methomyl and profenofos) to AChE reduces its activity, and the residual activity is monitored based on the yellow color intensity that is produced. Following this simple and reliable assay mechanism, we show that it is possible to detect the AChE inhibitors in a fast, disposable, cheap, and accurate format. The new biosensor detected methomyl and profenofos with detection limits of 6.16 × 10^−4^ mM and 0.27 mM, respectively, in 5 min incubation time. The operational stability of the biosensors was confirmed when glucose was used as stabilizer according to Kavruk et al. [30]. Hossain et al. [15] described a novel paper-based solid-phase biosensor that utilizes piezoelectric inkjet printing of biocompatible, enzyme-doped, sol-gel-based inks to create colorimetric sensor strips. The sensor consisted of AChE enzyme and indophenol acetate reagent for the assay where the combination of the two created a blue color. The presence of pesticides was indicated by a decrease in the blue color intensity which was interpreted using a digital camera. Although the analysis still requires additional processing steps using a computer, the sensor is still considered simple and rapid compared to other sophisticated laboratory instrumentation. They found that the assay provided good detection limits (paraoxon, ~0.1 mM; aflatoxin B1, ~0.03 mM) and rapid response times (<5 min). Kavruk et al. [30] developed a paper-based biosensor for the detection of the degradation products of organophosphorus pesticides. The new biosensor detected malathion with a detection limit of 5.57 × 10^−3^ mM.

Enzyme-based biosensors have been known for their selectivity, specificity, and catalytic signal amplification for the development of biosensors [30, 38, 39]. However, the applications of enzyme-based sensors remain limited mostly due to the liability for extreme physical conditions. Thus, special procedures are required for increasing their stability during storage. Immobilized enzymes can exhibit much better functional properties when immobilization strategies are properly designed for the enzyme [40]. In our biosensor, the immobilization and stability of the AChE is the most labile part of the biosensor. The activity loss of the enzyme should be controlled for a viable application of the biosensor. Therefore, AChE was crosslinked with biopolymer chitosan molecules by glutaraldehyde in the presence of DTNB and glucose as stabilizer [30, 41] and tested for the effect on the enzyme activity when immobilized in the paper.

## 5. Conclusion

In this work we have developed and performed a dipstick-type AChE inhibitor sensor for determining the optimum concentrations of sensor components. Depending on the immobilization method with a biopolymer chitosan, visual colorimetric has been applied as detection method and the paper-based biosensor has been tested on standards for the determination of two pesticides commonly used: methomyl and profenofos. The results showed a good performance of the developed sensors with reasonable limits of detection, as well as good reproducibility and stability. The new biosensor detected methomyl and profenofos with detection limits of 6.16 × 10^−4^ mM and 0.27 mM, respectively, in 5 min incubation time. Following this simple and reliable assay mechanism, we show that it is possible to detect the AChE inhibitors in a rapid and low cost effective manner.
